# Sex-based antibody subclass maturation drives direct enzymatic inhibition in fabry disease patients receiving enzyme replacement therapy

**DOI:** 10.3389/fmolb.2026.1702751

**Published:** 2026-04-09

**Authors:** Tomas Baldwin, Hibba Kurdi, Ivan Doykov, Francesca Robertson, Stefania Rosmini, Sabrina Nordin, Joao Augusto, Rebecca Kozor, Julien Baruteau, James Davison, David Moreno-Martinez, Uma Ramaswami, Ravi Vijapurapu, Tarekegn Geberhiwot, Rick Steeds, James Moon, Derralynn Hughes, Kevin Mills, Wendy E. Heywood

**Affiliations:** 1 Genetics and Genomic Medicine Department, UCL Institute of Child Health, London, United Kingdom; 2 Institute of Cardiovascular Science, University College London, London, United Kingdom; 3 Kings College London, London, United Kingdom; 4 University of Glasgow, Glasgow, United Kingdom; 5 Queen Elizabeth University Hospital, Glasgow, United Kingdom; 6 Royal North Shore Hospital, Sydney, NSW, Australia; 7 Sydney Medical School, University of Sydney, Sydney, NSW, Australia; 8 Great Ormond Street Hospital for Children NHS Foundation Trust, London, United Kingdom; 9 Royal Free London NHS Foundation Trust, London, United Kingdom; 10 Queen Elizabeth Hospital, University Hospitals Birmingham NHS Foundation Trust, Birmingham, United Kingdom; 11 St. Bartholomew’s Hospital, Barts Health NHS Trust, London, United Kingdom; 12 University College London, London, United Kingdom

**Keywords:** agalsidase alfa, agalsidase beta, antibody isotype, anti-drug antibodies, pegunigalsidase alfa, enzyme replacement therapy, fabry disease, immunogenicity

## Abstract

**Background:**

Enzyme replacement therapy (ERT) for Fabry disease can elicit anti-drug antibodies (ADAs) that may diminish efficacy and limit clinical benefit.

**Objectives:**

To develop and apply a multiplexed proteomic assay for quantitative, subclass-specific ADA and complement profiling in Fabry disease to inform personalized ERT selection.

**Methods:**

We created a targeted LC-MS/MS platform to quantify ADA binding across IgG1–4, IgM, and IgA1, and complement proteins C1Qc–C9, in serum from Fabry patients (n = 39) and healthy controls. Neutralizing capacity was measured via enzymatic inhibition assay. Subclass-specific cross-reactivity was assessed for agalsidase alfa, agalsidase beta, and pegunigalsidase alfa.

**Results:**

IgG4 binding was significantly higher in Fabry males (p = 0.007), with no sex-based differences for other Ig classes. Complement binding (C1Qc, C3) was elevated in ∼25% of patients, with IgG1, IgG2, IgM, and IgA1 correlating with C1Qc (r > 0.6). Seven patients (three female) exhibited >50% ERT inhibition; IgG4 binding correlated with enzymatic inhibition (p < 0.0025) and elevated lyso-Gb3 in males (p < 0.02). We assessed cross-reactivity of IgG4 in a patient who had received only agalsidase alfa, finding a 49% reduction in IgG4 binding to Pegunigalsidase alfa compared to Agalsidase alfa (p = 0.003) and 45% to Peguingalsidase alfa compared to agalsidase beta (p = 0.035). IgG4 comprised >50% of immune complexes for agalsidase alfa/beta but only 25% for pegunigalsidase alfa, indicating a potentially distinct immunogenic profile.

**Conclusion:**

Quantitative subclass-specific ADA and complement profiling reveals sex-specific IgG4 patterns, neutralizing capacity, and ERT-specific immunogenic differences, supporting its utility for personalized therapy in Fabry disease.

**Capsule summary:**

A novel multiplex LC-MS/MS assay quantifies ADA subclasses and complement in Fabry disease, uncovering distinct IgG4 patterns and ERT-specific profiles that enhance understanding of treatment immunogenicity.

## Introduction

Fabry disease (FD) is a rare X-linked lysosomal storage disorder caused by mutations in the *GLA* gene encoding alpha galactosidase A (AGAL), an enzyme responsible for degrading globotriaosylceramide (Gb3). Deficient or absent AGAL leads to progressive accumulation of Gb3 and its deacylated form lyso-Gb3 in multiple tissues, including kidney, heart, and nervous system, resulting in diverse manifestations from neuropathic pain (Burlina et al., 2011) to renal failure (Thadhani et al., 2002), cardiovascular disease (Schiffmann et al., 2009), and cerebrovascular events (Sims et al., 2009). While males are typically more severely affected, heterozygous females can also experience significant morbidity due to X-inactivation and DNA methylation (Echevarria et al., 2016).

Disease-modifying therapy includes enzyme replacement therapy (ERT), with three recombinant AGAL forms currently available in the UK: agalsidase alfa, agalsidase beta, and pegunigalsidase alfa. For patients with amenable mutations, oral chaperone therapy is also available. ERT reduces Gb3/lyso-Gb3 accumulation and improves outcomes (Germ et al., 2007; West et al., 2009), but effectiveness varies - particularly in males - and is greater when initiated before age 40 (Germain et al., 2015).

A key limitation of ERT is anti-drug antibody (ADA) development, especially in classical males with minimal residual enzyme activity (Arends et al., 2018; Linthorst et al., 2004). ADAs can neutralise enzyme activity, increase clearance, or limit uptake (Lenders et al., 2018), correlating with reduced impact on lyso-Gb3 and, in some cases, worse outcomes (Rombach et al., 2012; Lenders et al., 2016). Adverse infusion related reactions can also occur and need to be monitored (Barbey and Livio, 2006). ADA subclass and isotype influence neutralising potential, with IgG1 and IgG4 more likely to mediate these effects (Lenders et al., 2018; van der Veen et al., 2019). Understanding these immune responses is critical for optimising therapy.

To further our understanding of how antibody subclass and isotype response impacts therapeutic efficacy in patients receiving ERT in FD, we have adapted an antibody analysis platform developed during the SARS-CoV-2 pandemic. This ‘bait and capture’ methodology, coupled with multiplexed and targeted proteomic liquid chromatography-tandem mass spectrometry (LC-MS/MS) analyses, allows for simultaneous quantitation of all key components of the immune complex including immunoglobulins, complement and bait antigen (Doykov et al., 2022). In this study, we utilise this novel platform to investigate immune complex formation *in vitro* against ERT in male and female patients with FD. Our results indicate that individual subclass analysis could be important in determining whether a patient is at risk of developing neutralising antibodies (nAbs), and that utilisation of this platform could aid in providing a personalised medicine approach to therapeutic strategies.

## Methods

### Materials

Agalsidase alfa was received as a gift from the Royal Free Hospital. Agalsidase beta was received as a gift from Great Ormond Street Hospital. Pegunigalsidase alfa was provided by Chiesi limited for academic research upon request. Ninety six-well deep well (700 μL) plates were purchased from Waters Corporation (Wilmslow, UK). Unless specifically stated, all reagents were purchased from Merck (Gillingham, UK). QConCat internal standard was purchased from PolyQuant GmbH (Germany).

### Ethics statement and cohort characteristics

The study complied with the Declaration of Helsinki and obtained approval from the local research ethics committee (REC reference: 07/H0715/101). Healthy recruited volunteers were free of cardiovascular disease and diabetes, although a small number took statins for primary prevention, and all had normal electrocardiograms. FD participants were enrolled from four FD clinics as part of the prospective, multicentre, international Fabry400 observational study (NCT03199001) (Table 1). Eligible FD patients were gene-positive and 18 years or older, while healthy volunteer controls had no history of cardiovascular disease (as verified by a normal health questionnaire) and were not on cardioactive medication except for primary prevention. Standard contraindications to CMR constituted exclusion criteria. Additionally, one patient was included from the Study of Inherited Metabolic Disease (SIMD) database, 13/LO/0168, IRAS project ID: 95005.

**TABLE 1:** Cohort data used in this study.

Cohort characteristics	
Fabry (n)	39
Control (n)	31
Sex, male (%)	48%
Age in years, median (range)	48.5 (16–73)
Receiving ERT (%)	52%
Agalsidase alfa (%)	60%

### Anti-alpha-galactosidase A immune complex assay

To determine serum immune complex formation against recombinant alphagalactosidase A (AGAL) a previous assay was adapted from the method developed in Doykov et al., 2022. 96-well plates were coated with agalsidase alfa, agalsidase beta, or pegunigalsidase alfa (50 μg/mL PBS; 10 µL/well) plus 140 µL 100 mM carbonate/bicarbonate buffer (pH 9.6) and incubated 16 h at 4 °C. Plates were washed, blocked (1 mg/mL horse myoglobin/PBS, 1 h, RT), washed, and stored at 4 °C. Serum (1:10 in 0.05 mg/mL myoglobin/PBS; 150 µL/well) was incubated 1 h at 37 °C, washed (0.05% Tween 20/PBS, then PBS ×3), dried by centrifugation, and stored at 4 °C.

### Protein digestion

Bound proteins were resuspended in 70 µL 0.5% DOC/50 mM AmBic with 10 µL 1 μg/mL QConCat. DTT (162 mM; 3 µL) was added (85 °C, 20 min, 750 rpm), cooled, treated with IAA (162 mM; 6 μL, 30 min, dark), digested with trypsin (5 μL; 1 mg/mL, 45 °C, 30 min), and quenched with 5 µL 6% TFA. Plates were centrifuged (4,000 g, RT, 30 min) and 50 µL supernatant collected for LC-MS/MS.

### Targeted LC-MS/MS

Peptides were separated on a Waters UPLC Premier C18 (1.7 µm, 2.1 × 50 mm, 45 °C) at 0.3 mL/min with 0.1% formic acid in water (A) and ACN (B): 5% B (0.1 min), 5%–40% B (7.7 min), 40%–80% B (0.2 min), hold 1 min, re-equilibrate 1 min. Detection was on a Xevo-TQ-XS in positive ESI MRM mode (capillary 2.8 kV, source 150 °C, desolvation 600 °C, cone/desolvation gas 150/800 L h^-1^, N_2_ collision gas 0.15 mL/min, cone voltage 35 V, peptide-specific collision energies).

## Biochemical analyses

### ERT inhibition assay

As described in (Lenders et al., 2016), (Desnick et al., 1973), 5 µL serum was incubated 10 min at RT with 1 ng ERT (agalsidase alfa, agalsidase beta, or pegunigalsidase alfa). Activation solution (100 μL; 3.5 mM 4-methylumbelliferyl-α-d-galactopyranoside, 5 mM N-acetylgalactosamine, 100 mM citrate, 200 mM phosphate, pH 4.6) was added, and samples incubated 60 min at 37 °C. Reactions were stopped with 300 mM glycine buffer (pH 10.6). ERT activity was expressed as % of uninhibited control (1 ng ERT). Samples were run in duplicate; results are mean ± SD.

### Plasma Lyso-Gb3 quantification by LC-MS/MS

Adapted from (Heywood et al., 2019), lyso-GB3 was extracted from 100 µL plasma with 1 mL acetone:methanol (1:1 v/v) containing 2 ng/mL N-glycinated lyso-ceramide trihexoside (Matreya, LLC) as internal standard. Samples were shaken 20 min, sonicated 15 min, shaken 20 min, centrifuged (16,000 × g, RT), and supernatants evaporated. Residues were reconstituted in 100 µL methanol for LC-MS/MS (UPLC Xevo-TQ-S, Waters). MRM detection was in positive ESI mode. Mobile phases: A = 0.1% formic acid in water; B = 100% methanol; flow rate 0.6 mL/min.

### Data and statistical analysis

Raw LC–MS/MS data were processed in Skyline (MacLean et al., 2010). Two optimal transitions were selected for final MRM analysis, with confirmation via heavy peptides from a custom QConCAT standard. Peptide abundance was normalised to an αGalA-specific peptide (SILDWTSFNQER) to calculate antibody per ng ERT bait. Statistics were performed in GraphPad Prism v9.5: Mann–Whitney for pairwise comparisons, Kruskal–Wallis for groups; p < 0.05 was considered significant.

## Results

### Generation of fully quantitative reference ranges for anti-drug antibody binding

**FIGURE 1:**
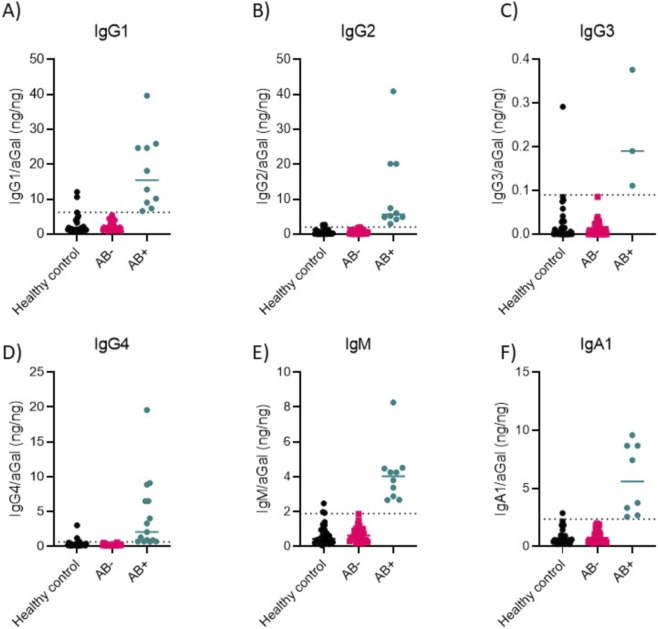
Proposed quantitative reference ranges for antibody subclass and isotype In FD adults using targeted LC-MS/MS. 97.fifth percentile reference ranges were generated using healthy negative control serum. These values were then applied to **(A)** IgG1, **(B)** IgG2, **(C)** IgG3, **(D)** IgG4, **(E)** IgM and **(F)** IgA1, which were then used to generate positive (AB+) and negative (AB-) groupings for FD patient samples. N = 30 healthy controls and n = 39 FD patients. Positive threshold >97.fifth percentage indicated by dotted line. N = 30 Healthy control and n = 39 FD.

To establish robust thresholds for classifying immune responses, 97.fifth percentile reference ranges were generated using serum from healthy control donors in nanogram of antibody per nanogram of ERT bait. These cutoffs were then applied to FD patient samples to determine positivity or negativity for each immunoglobulin subclass and isotype, namely, IgG1, >6.29 ng/ng (Figure 1A), IgG2, >2.06 ng/ng (Figure 1B), IgG3, >0.0805 ng/ng (Figure 1C), IgG4, >0.58 ng/ng (Figure 1D), IgM, >1.98 ng/ng (Figure 1E), and IgA1, >2.36 ng/ng (Figure 1F). This approach enabled a standardized evaluation of antibody levels in the FD cohort and facilitated the identification of potential immunoglobulin reactivity patterns across different subclasses and isotypes. The greatest responses for each subclass and isotype were observed as 39.66 ng/ng for IgG1 (Figure 1A), 40.88 ng/ng IgG2 (Figure 1B), 0.39 ng/ng IgG3 (Figure 1C), 19.57 ng/ng IgG4 (Figure 1D), 8.23 ng/ng IgM (Figure 1E), and 9.48 ng/ng IgA1 (Figure 1F).

### Sex based differences in ADA binding in FD patients

**FIGURE 2:**
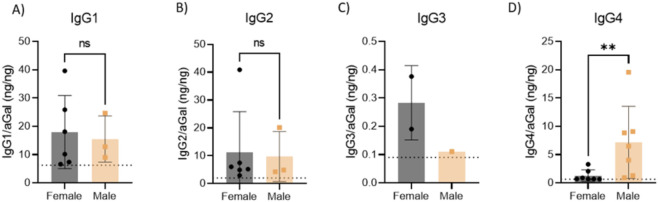
Analysis of positive responses in males and females suggests subclass-based differences. Utilising the previously defined reference ranges, we observed no sex-based differences in positive responses when assessing **(A)** IgG1, **(B)** IgG2 or **(C)** IgG3. However, levels of IgG4 binding in positive patients was observed to be significantly different (p = 0.007, n = 7 per group). Data are expressed as mean ± SD. Statistical significance determined by non-parametric T-Test (Mann-Whitney), results considered significant if p < 0.05, ** represents p < 0.001.

An examination of positive responses in males and females indicated potential differences by subclass. Based on previously established reference ranges, no sex-based differences were observed in IgG1 (Figure 2A), IgG2 (Figure 2B), or IgG3 (Figure 2C) binding. However, IgG4 levels in positive patients differed significantly between males and females (Figure 2D; p < 0.007), implying that IgG4 may be distinctly regulated in each sex. Thirty eight percent of males were determined to be positive for an IgG4 response, matching previously described IgG responses (Lenders and Brand, 2018).

### Analysis of associated complement binding to ADA complex

**FIGURE 3:**
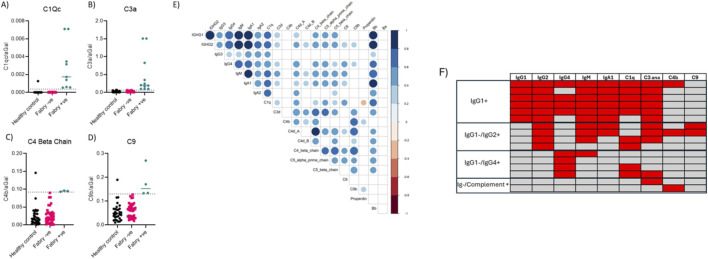
Analysis of complement binding to ADA immunocomplex. Binding of complement components from C1Qc through to C9 was assessed and 97.fifth percentile reference ranges were generated using healthy control serum. **(A)** Binding of C1QC and **(B)** C3 was observed in approximately 25% of FD patients, and only one healthy control sample. Whereas a positive binding response of later components of the complement cascade was observed in >10% of FD patients **(C,D)**. **(E)** FD patient data indicates that there is a strong positive correlation between IgG1, IgG2, IgM and IgA1 binding levels and complement–particularly C1Qc. Significance determined by Pearson correlation; results considered significant if p < 0.05. **(F)** Heat map showing distribution of components of the immune complex in positive samples. N = 30 healthy control, n = 39 FD disease.

Binding of complement components C1Qc through C9 was assessed in the immune complexes from healthy controls and patients with FD. Reference ranges were established for each component based on the 97.fifth percentile of healthy control serum. C1Qc (Figure 3A) and C3 (Figure 3B) binding was observed in approximately 25% of FD patients but in only one control sample. Spearman’s correlation analyses in healthy control samples revealed no significant relationship between ADA binding and any complement component; however, a strong positive correlation was noted among the complement proteins themselves (Supplementary Figure S1). In FD patients, binding levels of IgG1, IgG2, IgM, and IgA1 showed a strong positive correlation with complement proteins, particularly C1Qc (Figure 3E). When observing the bound immune complex collectively in the heat map (Figure 3F) we observe that 43% of total IgG positive samples (n = 14) are positive for IgG1 with 72% positive for IgG2 and 57% positive for IgG4. The majority of IgG1+ samples were also positive for other components of the immune complex including IgM, IgA1 C1q and C3. A subgroup of samples (n = 4) were positive only for IgG2 and another subgroup (n = 4) positive only for IgG4 which did not have any association with IgA1. We did detect 2 samples which had an independent complement response (C3 or C4) with no immunoglobulin response which is indicative of alternative complement activation. Whilst we did not see an IgA only response in this particular cohort of samples we did observe in a previous analysis a subgroup of IgA only response patients (Supplementary Figure S2).

### Correlation between IgG4 binding and direct enzymatic inhibition in FD males

**FIGURE 4:**
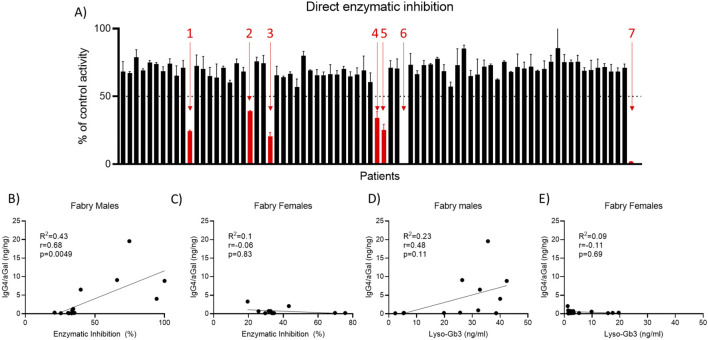
Assessing cross reactivity of neutralising IgG4 signal. **(A)** FD male patient’s serum neutralising capacity was assessed against agalsidase alfa, beta and pegunigalsidase alfa. **(B)** Patient IgG4 binding assessed against each enzyme, with binding against pegunigalsidase alfa significantly lower (p = 0.003) than binding against agalsidase alfa and beta (p = 0.034). Assessing patient total immune complex against **(C)** agalsidase alfa **(D)** beta and **(E)** pegunigalsidase alfa indicates a relationship between binding against agalsidase alfa and beta (IgG4 >50% total) compared to pegunigalsidase alfa (IgG4 25%). n = 14 FD males. Statistical significance determined by non-parametric ANOVA (Kruskal–Wallis), results considered significant if p < 0.05.

The effect of patient serum on ERT activity was evaluated, with a reduction of activity below 50% of control defining a neutralizing response (Lenders et al., 2016). Using this cutoff, seven FD patients were identified with neutralizing antibodies (nAbs), three of whom were female, which has not been observed previously (Figure 4A). Further analysis revealed a strong positive correlation between IgG4 binding and direct enzymatic inhibition (Figure 4B; p = 0.0025). This association was not observed in female patients (Figure 4C; p = 0.83). There was no observed relationship between direct enzymatic inhibition levels of other antibody subclasses or isotypes (Supplementary Figure S3). Additionally, IgG4 binding was associated with elevated plasma lyso-Gb3 levels, however this did not reach statistical significance (Figure 4D; p = 0.11). Time from start of ERT could explain this observation as IgG4 evolves over time from repeat exposure. A relationship again was absent among female patients (Figure 4E; p = 0.69). Similarly, we found no relationship between plasma lyso-Gb3 levels in males and other antibody subclasses and isotypes.

### Pre-existing neutralizing antibodies against agalsidase alfa and beta show reduced inhibitory activity against pegunigalsidase alfa

**FIGURE 5:**
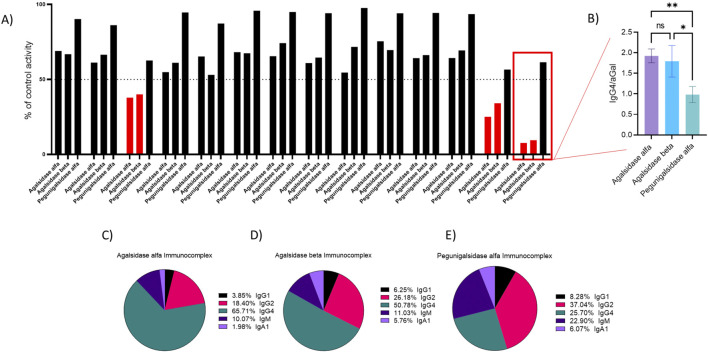
Relationship between ADA and direct enzymatic inhibition. **(A)** Effect of patient serum on ERT activity was assessed. Reduction of activity below 50% of control was deemed to be neutralising. 7 FD patients were identified with neutralising antibodies (nAbs) (Indicated with red arrows). 3 of these patients were female (Burlina et al., 2011; Thadhani et al., 2002; Schiffmann et al., 2009). **(B)** Strong positive correlation (p = 0.0025) observed between IgG4 binding and direct enzymatic inhibition. **(C)** This was not observed in females (p = 0.83). **(D)** IgG4 binding also associated with increased plasma lyso-Gb3 in FD males, however this did not reach statistical significance (p = 0.11). **(E)** This trend was not observed in female patients (p = 0.69). n = 42 FD disease, n = 37 Healthy control. Statistical significance determined by Pearson’s correlation.

We next evaluated whether the neutralising IgG4 response elicited by agalsidase alfa also inhibited agalsidase beta and pegunigalsidase alfa (Figure 5A). We observe a consistent relationship between percentage inhibition of agalsidase alfa and beta, including the three patients determined to have a neutralising serum response to the enzymes (>50% inhibition). However, for each of these patients, there was a reduction of the serum inhibitory capacity against pegunigalsidase alfa, such that all three patients were brought above levels where inhibition was determined to be neutralising (<50% inhibition). Further, there was a consistently lower level of enzymatic inhibition of pegunigalsidase alfa across the group, including patients determined not to have a fully neutralising response against agalsidase alfa and beta. Analysis of IgG4 binding revealed that serum binding to pegunigalsidase alfa was significantly lower compared to agalsidase alfa (p = 0.003) and beta (p = 0.0349; Figure 5B). Further assessment of total immune complex formation against agalsidase alfa (Figure 5C), agalsidase beta (Figure 5D), and pegunigalsidase alfa (Figure 5E) indicated that IgG4 accounted for more than half of the total immune complex in samples tested against agalsidase alfa and beta (>50%), whereas for pegunigalsidase alfa, IgG4 comprised only 25%, replaced in the immune complex by IgM (Figure 5E). This reduced IgG4 contribution in complexes with pegunigalsidase alfa suggests a distinct immunogenic profile for this particular ERT, which may contribute to the reduced impact on enzymatic activity against this enzyme. The relationship between this anti-ERT IgG4 and each enzyme was demonstrated to be linear against all three enzymes (Supplementary Figure S4) for one of the positive males, highlighting the specificity of the response.

## Discussion

Currently, clinical anti-drug antibody (ADA) testing for Fabry disease in the United Kingdom is conducted almost exclusively by, or on behalf of, the pharmaceutical companies that manufacture enzyme replacement therapies (ERTs), rather than by independent laboratories. As a result, clinicians and researchers have limited access to cross-reactivity data, which is critical for understanding whether antibodies generated against one ERT also recognize other available products. This lack of standardization hinders the comprehensive assessment of immune responses, especially when patients switch therapies or are involved in head-to-head treatment comparisons.

A further challenge lies in the variability of methodologies used across laboratories. Most labs employ different techniques, often based on ELISA, which leads to inconsistent reproducibility and long turnaround times that are unsuitable for clinical decision-making. Moreover, this methodological inconsistency complicates the interpretation of ADA titers, as a single numerical value can be misleading without context. The quantitative, detailed nature of the methodology presented here aims to address this gap by offering a more standardized and interpretable framework, improving both clinical utility and scientific comparability.

This methodology provides several advantages over traditional ADA testing methods. The outputs of the mass spectrometry-based assay provide absolute quantitation in nanogram of antibody binding to nanogram of ERT, have low CV between replicates (typically <10%), can normalise intra-well to the ERT ‘bait’ signal, and can simultaneously quantitate binding of all antibody subclasses, isotypes and associated complement (van der Veen et al., 2019). Our first objective was to generate fully quantitative reference ranges for six immunoglobulin subclasses and isotypes, enabling a standardized approach to detecting ADA positivity in FD patients. Utilising 97.fifth percentile cutoffs generated from assessing ADA binding in a cohort of healthy control volunteers, we established individual reference ranges for IgG1, IgG2, IgG3, IgG4, IgM and IgA1. We also sought to generate reference ranges for IgA2 and IgE, however, these were both below the lower limit of quantitation in this cohort. Positivity for individual isotypes ranged from 16% for IgG1 and IgG2, to 38% for IgG4 in males, aligning with previously reported data for existing assays (Lenders and Brand, 2018).

The observation of higher IgG4 binding in male patients compared with females is therefore consistent with the well-described association between IgG4 and neutralising anti-drug antibody formation in Fabry disease (Lenders et al., 2018). Within this established framework, our data support the relevance of IgG4 in the humoral response to ERT while extending existing observations through immune complex–based profiling. Notably, IgG4 has been associated with immune regulation in other conditions (Rispens and Huijbers, 2023), yet here appears to confer deleterious, neutralizing properties toward AGAL. This phenomenon has been observed in other studies and strengthens the argument for IgG4 playing a significant role in the reduction of ERT efficacy in male FD patients (Lenders et al., 2018). The strong correlation observed between IgG4 binding, direct enzymatic inhibition and association with elevated plasma lyso-Gb3 in male patients further underlines the clinical impact of this subclass on disease progression. A neutralising effect (>50% inhibition) was only observed in males when IgG4 binding exceeded 4 ng antibody per ng of enzyme, further work will aim to investigate the utility of these values as predictive cutoffs for neutralising capacity of a patient’s immune response. Whilst none of the other immunoglobulin subclasses or isotypes correlated with direct enzymatic inhibition, they may interfere with therapeutic efficacy through other mechanisms, such as cellular uptake inhibition or accelerated clearance, which were not assessed here. Further work will aim to investigate this with larger cohorts of patients. Three of the male patients presenting with a positive IgG4 signal were derived from patients with a deletion mutation, highlighting a link between the presence or absence of endogenous enzyme, and immune response, as described in (van der Veen et al., 2020). The lack of these associations in female patients indicates that cross reactive immunological material (CRIM) status influence the immunologic landscape in FD. Whilst no antibody response was associated with enzymatic neutralisation in the female cohort, 3 patients were identified with a serum neutralising capacity >50%, suggesting a non-antibody mediated neutralisation. Further investigation of these patients involving discovery proteomics will aim to interrogate other binding factors that appear to impact enzymatic activity in these patients.

Our platform enables us to also study complement deposition, which could give insight into the type of immune response and potentially adverse events. Complement binding was assessed via C1Qc through C9 binding. A positive response for C1Qc and C3 binding was observed in approximately 25% of FD patients. Of particular interest, IgG1, IgG2, IgM, and IgA1 binding levels showed strong positive correlations with C1Qc in FD patients, potentially indicating that immune complexes involving these antibody classes trigger the classical complement pathway. However, the lack of significant correlations between ADA binding and complement proteins in healthy controls suggests that patients with FD disease may experience a more pronounced, disease-specific immune activation. Complement activation has been demonstrated to correlate with type-II hypersensitivity reactions in patients receiving ERT, however, it is suggested to not be the predominant cause of infusion related events in FD (Limgala et al., 2020). Further, a recent study has demonstrated constitutively elevated complement activation in classical males both before and after receiving ERT (Laffer et al., 2024). This work also demonstrated increased levels of circulating IL-6, IL-10 and TGF-β, hypothesising that this chronic state of complement activation and inflammation may drive progressive organ damage. However, it has been hypothesised that these ERT/ADA complexes may lead to complement deposition in tissue, worsening clinical progression, as has been observed in Pompe disease (Hunley et al., 2004). Taken together, there is a commonly observed correlation between complement activation and disease progression, but more work is needed to investigate the extent to which ERT/ADA/complement immune complexes may contribute to this. In this study, immune complexes were generated *in vitro* by incubating patient serum with recombinant ERT, enabling quantitative assessment of antibody subclass and complement binding capacity. This approach does not directly demonstrate the presence of circulating immune complexes *in vivo*. The frequency, composition, and clinical relevance of circulating ERT–ADA complexes in treated patients therefore remain to be determined and warrant dedicated investigation in future studies.

In a pilot study, we assessed two female patients - mother and daughter–presenting with abdominal disturbances either during or post-infusion with ERT. When profiling their antibody response, whilst usually undetectable, only IgA2 was observed to be increased. This was associated with a C1Q independent activation and deposition of the terminal complement cascade (Supplementary Figure S5). IgA2 is the predominant antibody in the intestinal mucosa (Cerutti, 2008), and our data suggest that IgA2 targeting of ERT in the intestine may lead to complement activation and deposition, leading to these observed intestinal disturbances.

An additional emerging observation is the presence of free circulating inhibitory ADAs and their impact on pharmacokinetics, particularly in patients treated with pegunigalsidase alfa. The clinical relevance of these circulating ADAs, and the potential formation of immune complexes, has been demonstrated previously, correlating with significant loss of eGFR in ADA positive patients (Holida et al., 2025). The detection of both free inhibitory ADAs and immune complex formation in this study underscores the need for future work to more fully characterise immune complex dynamics and their clinical consequences. Future work will aim to establish the frequency of these complexes in patients treated with pegunigalsidase alfa and to develop approaches to decouple and analyse the antibody and complement components.

A limitation of the present study is that antibody subclass profiling was performed at a single timepoint for each patient. While the observed patterns of IgG and IgA subclass engagement and complement association are most consistent with antigen-specific recognition of recombinant AGAL, longitudinal sampling would further strengthen causal inference. Future studies will incorporate consecutive measurements within individual patients, including baseline, early treatment, and extended follow-up timepoints, as well as post-switch analyses where applicable. These longitudinal profiling will enable direct assessment of antibody class switching and maturation (e.g., IgG1/IgG3 to IgG4), the temporal relationship between immune complex formation and clinical symptoms, and the stability of subclass-specific binding over time.

In this study, we demonstrate that we have developed a method that can not only quantitate antibody response associated with efficacy but also quantify complement reactions associated with ERT – and potentially even the route of activation. The finding that pegunigalsidase alfa elicits lower IgG4 binding than agalsidase alfa or beta provides critical insight into the immunogenic profiles of these ERTs. In one highly neutralizing male patient who had received only agalsidase alfa, pegunigalsidase alfa exhibited significantly reduced IgG4-mediated immune complex formation, accounting for only 25% of the total complex compared to more than 50% in agalsidase alfa and beta complexes. This finding was observed in all male patients who presented with a neutralising response against agalsidase alfa. This reduction translates into diminished neutralizing activity against pegunigalsidase alfa, potentially improving therapeutic efficacy and long-term clinical outcomes in patients prone to IgG4-mediated nAb development. These findings also mirror previous work indicating that there is a diminished level of cross-reactivity for pre-existing nAbs (Lenders et al., 2022), but goes further to demonstrate that this extends to the IgG4 subclass. However, whilst we observe less cross reactivity of IgG we did find increased IgM binding which may indicate an individual immune response to pegunigalsidase alfa could occur if administered. Further evaluation of patients who have switched to pegunigalsidase alfa is needed to see if the more specific IgG develop on from the IgM response. This approach enables an integrated evaluation of a patient’s immune response to all three commercially available enzymes, thereby allowing for more informed therapeutic decisions. However, continual monitoring of immune complex formation/alterations remains essential to detect any emerging ADA responses that could compromise treatment efficacy or trigger complement activation.

Collectively, these results underscore the necessity of robust immunogenicity assessments in FD management. Establishing reliable reference ranges for ADAs, understanding sex-specific immunologic patterns, and elucidating ERT-specific immunogenic profiles can inform individualized treatment strategies. Future studies should further explore the mechanisms governing IgG4 regulation in FD, investigate immune modulation approaches to mitigate nAb formation, and evaluate the long-term clinical impact of utilizing pegunigalsidase alfa in patients with high-titer IgG4 responses.

## Limitations

There are some limitations to the study detailed here. Firstly, Outputs of our LC-MS/MS assay were only correlated against direct enzymatic inhibition and peripheral biomarkers (lyso-Gb3). *In vivo*, ADA can also impact therapeutic efficacy through other mechanisms, such as cellular uptake inhibition and accelerated clearance. Relationships between antibody isotype, complement deposition and these mechanisms can be explored in future studies. Secondly, as we only assess a single timepoint per patient, we are capturing a snapshot of each patients immune complex response to ERT. Future work will investigate longitudinal changes in the immune complex for patients switching treatment.

## Data Availability

Data is available upon reasonable request to the authors.

## References

[B1] Arends M. Biegstraaten M. Wanner C. Sirrs S. Mehta A. Elliott P. M. (2018). Agalsidase alfa *versus* agalsidase beta for the treatment of fabry disease: an international cohort study. J. Med. Genet. 55 (5), 351–358. 10.1136/jmedgenet-2017-104863 29437868 PMC5931248

[B2] Barbey F. Livio F. (2006). Safety of enzyme replacement therapy. 21290709

[B3] Burlina A. P. Sims K. B. Politei J. M. Bennett G. J. Baron R. Sommer C. (2011). Early diagnosis of peripheral nervous system involvement in Fabry disease and treatment of neuropathic pain: the report of an expert panel. BMC Neurol. 11, 61. 10.1186/1471-2377-11-61 21619592 PMC3126707

[B4] Cerutti A. (2008). The regulation of IgA class switching. Nat. Rev. Immunol. 8, 421–434. 10.1038/nri2322 18483500 PMC3062538

[B5] Desnick R. J. Allen K. Y. Desnick S. J. Raman M. K. Bernlohr R. W. Krivit W. (1973). Fabry’s disease: enzymatic diagnosis of hemizygotes and heterozygotes. Alpha-galactosidase activities in plasma, serum, urine, and leukocytes. J. Lab. Clin. Med. 81 (2), 157–171. 4683418

[B6] Doykov I. Baldwin T. Spiewak J. Gilmour K. C. Gibbons J. M. Pade C. (2022). Quantitative, multiplexed, targeted proteomics for ascertaining variant specific SARS-CoV-2 antibody response. Cell Rep. Methods 2 (9), 100279. 10.1016/j.crmeth.2022.100279 35975199 PMC9372021

[B7] Echevarria L. Benistan K. Toussaint A. Dubourg O. Hagege A. A. Eladari D. (2016). X-chromosome inactivation in female patients with Fabry disease. Clin. Genet. 89 (1), 44–54. 10.1111/cge.12613 25974833

[B8] Germain D. P. Waldek S. Banikazemi M. Bushinsky D. A. Charrow J. Desnick R. J. (2007). Sustained, long-term renal stabilization after 54 months of agalsidase beta therapy in patients with Fabry disease. J. Am. Soc. Nephrol. 18 (5), 1547–1557. 10.1681/ASN.2006080816 17409312

[B9] Germain D. P. Charrow J. Desnick R. J. Guffon N. Kempf J. Lachmann R. H. (2015). Ten-year outcome of enzyme replacement therapy with agalsidase beta in patients with Fabry disease. J. Med. Genet. 52 (5), 353–358. 10.1136/jmedgenet-2014-102797 25795794 PMC4413801

[B10] Heywood W. E. Doykov I. Spiewak J. Hallqvist J. Mills K. Nowak A. (2019). Global glycosphingolipid analysis in urine and plasma of female Fabry disease patients. Biochimica Biophysica Acta (BBA) - Mol. Basis Dis. 1865 (10), 2726–2735. 10.1016/j.bbadis.2019.07.005 31319156

[B11] Holida M. Linhart A. Pisani A. Longo N. Eyskens F. Goker-Alpan O. (2025). A phase III, open-label clinical trial evaluating pegunigalsidase alfa administered every 4 weeks in adults with Fabry disease previously treated with other enzyme replacement therapies. J. Inherit. Metab. Dis. 48 (1), e12795. 10.1002/jimd.12795 39381863 PMC11667655

[B12] Hunley T. E. Corzo D. Dudek M. Kishnani P. Amalfitano A. Chen Y. T. (2004). Nephrotic syndrome complicating α-Glucosidase replacement therapy for pompe disease. Pediatrics 114 (4), e532–e535. 10.1542/peds.2003-0988-L 15466083

[B13] Laffer B. Lenders M. Ehlers-Jeske E. Heidenreich K. Brand E. Köhl J. (2024). Complement activation and cellular inflammation in Fabry disease patients despite enzyme replacement therapy. Front. Immunol. 15, 1307558. 10.3389/fimmu.2024.1307558 38304433 PMC10830671

[B14] Lenders M. Brand E. (2018). Effects of enzyme replacement therapy and Antidrug antibodies in patients with fabry disease. J. Am. Soc. Nephrol. 29 (9), 2265–2278. 10.1681/ASN.2018030329 30093456 PMC6115664

[B15] Lenders M. Stypmann J. Duning T. Schmitz B. Brand S. M. Brand E. (2016). Serum-Mediated inhibition of enzyme replacement therapy in fabry disease. J. Am. Soc. Nephrol. 27 (1), 256–264. 10.1681/ASN.2014121226 25933799 PMC4696578

[B16] Lenders M. Schmitz B. Brand S. M. Foell D. Brand E. (2018). Characterization of drug-neutralizing antibodies in patients with Fabry disease during infusion. J. Allergy Clin. Immunol. 141 (6), 2289–2292.e7. 10.1016/j.jaci.2017.12.1001 29421273

[B17] Lenders M. Pollmann S. Terlinden M. Brand E. (2022). Pre-existing anti-drug antibodies in Fabry disease show less affinity for pegunigalsidase alfa. Mol. Ther. Methods Clin. Dev. 26, 323–330. 10.1016/j.omtm.2022.07.009 35990747 PMC9379515

[B18] Limgala R. P. Fikry J. Veligatla V. Goker-Alpan O. (2020). The interaction of innate and adaptive immunity and stabilization of mast cell activation in management of infusion related reactions in patients with fabry disease. Int. J. Mol. Sci. 21 (19). 10.3390/ijms21197213 33003611 PMC7583043

[B19] Linthorst G. E. Hollak C. E. M. Donker-Koopman W. E. Strijland A. Aerts JMFG (2004). Enzyme therapy for Fabry disease: neutralizing antibodies toward agalsidase alpha and beta. Kidney Int. 66 (4), 1589–1595. 10.1111/j.1523-1755.2004.00924.x 15458455

[B20] MacLean B. Tomazela D. M. Shulman N. Chambers M. Finney G. L. Frewen B. (2010). Skyline: an open source document editor for creating and analyzing targeted proteomics experiments. Bioinformatics 26 (7), 966–968. 10.1093/bioinformatics/btq054 20147306 PMC2844992

[B21] Rispens T. Huijbers M. G. (2023). The unique properties of IgG4 and its roles in health and disease. Nat. Rev. Immunol. 23 (11), 763–778. 10.1038/s41577-023-00871-z 37095254 PMC10123589

[B22] Rombach S. M. Aerts JMFG Poorthuis BJHM Groener J. E. M. Donker-Koopman W. Hendriks E. (2012). Long-term effect of antibodies against infused alpha-galactosidase A in Fabry disease on plasma and urinary (lyso)Gb3 reduction and treatment outcome. PLoS One 7 (10), e47805. 10.1371/journal.pone.0047805 23094092 PMC3477102

[B23] Schiffmann R. Warnock D. G. Banikazemi M. Bultas J. Linthorst G. E. Packman S. (2009). Fabry disease: progression of nephropathy, and prevalence of cardiac and cerebrovascular events before enzyme replacement therapy. Nephrol. Dial. Transpl. 24 (7), 2102–2111. 10.1093/ndt/gfp031 19218538 PMC2698092

[B24] Sims K. Politei J. Banikazemi M. Lee P. (2009). Stroke in Fabry disease frequently occurs before diagnosis and in the absence of other clinical events: natural history data from the Fabry Registry. Stroke 40 (3), 788–794. 10.1161/STROKEAHA.108.526293 19150871

[B25] Thadhani R. Wolf M. West M. L. Tonelli M. Ruthazer R. Pastores G. M. (2002). Patients with Fabry disease on dialysis in the United States. Kidney Int. 61 (1), 249–255. 10.1046/j.1523-1755.2002.00097.x 11786107

[B26] van der Veen S. J. van Kuilenburg A. B. P. Hollak C. E. M. Kaijen P. H. P. Voorberg J. Langeveld M. (2019). Antibodies against recombinant alpha-galactosidase A in Fabry disease: subclass analysis and impact on response to treatment. Mol. Genet. Metab. 126 (2), 162–168. 10.1016/j.ymgme.2018.11.008 30473480

[B27] van der Veen S. J. Hollak C. E. M. van Kuilenburg A. B. P. Langeveld M. (2020). Developments in the treatment of Fabry disease. J. Inherit. Metab. Dis. 43, 908–921. 10.1002/jimd.12228 32083331 PMC7540041

[B28] West M. Nicholls K. Mehta A. Clarke J. T. R. Steiner R. Beck M. (2009). Agalsidase alfa and kidney dysfunction in Fabry disease. J. Am. Soc. Nephrol. 20 (5), 1132–1139. 10.1681/ASN.2008080870 19357250 PMC2678048

