# Supplementary material for: Sex-based antibody subclass maturation drives direct enzymatic inhibition in fabry disease patients receiving enzyme replacement therapy
Source: Front Mol Biosci. 2026 Apr 9;13:1702751. doi: 10.3389/fmolb.2026.1702751 (PMC13102577; doi:10.3389/fmolb.2026.1702751)
Supplement: Supplementary file 1 [file DataSheet1.pdf]

Sex-based antibody subclass maturation drives direct enzymatic inhibition in Fabry patients receiving enzyme replacement therapy

Tomas Baldwin[1], Hibba Kurdi[2] Ivan Doykov[1], Francesca Robertson[1] ,Stefania Rosmini[3], Sabrina Nordin[4,5], Joao Augusto[2], Rebecca Kozor[5,6], Julien Barteau[1,8], James Davison[8], David Moreno-Martinez[9], Uma Ramaswami[9], Ravi Vijapurapu[10], Tarekegn Geberhiwot[10], Rick Steeds[10], James Moon[2,11], Derralynn Hughes[9], Kevin Mills[1], Wendy E. Heywood[1]

Supplementary Figures

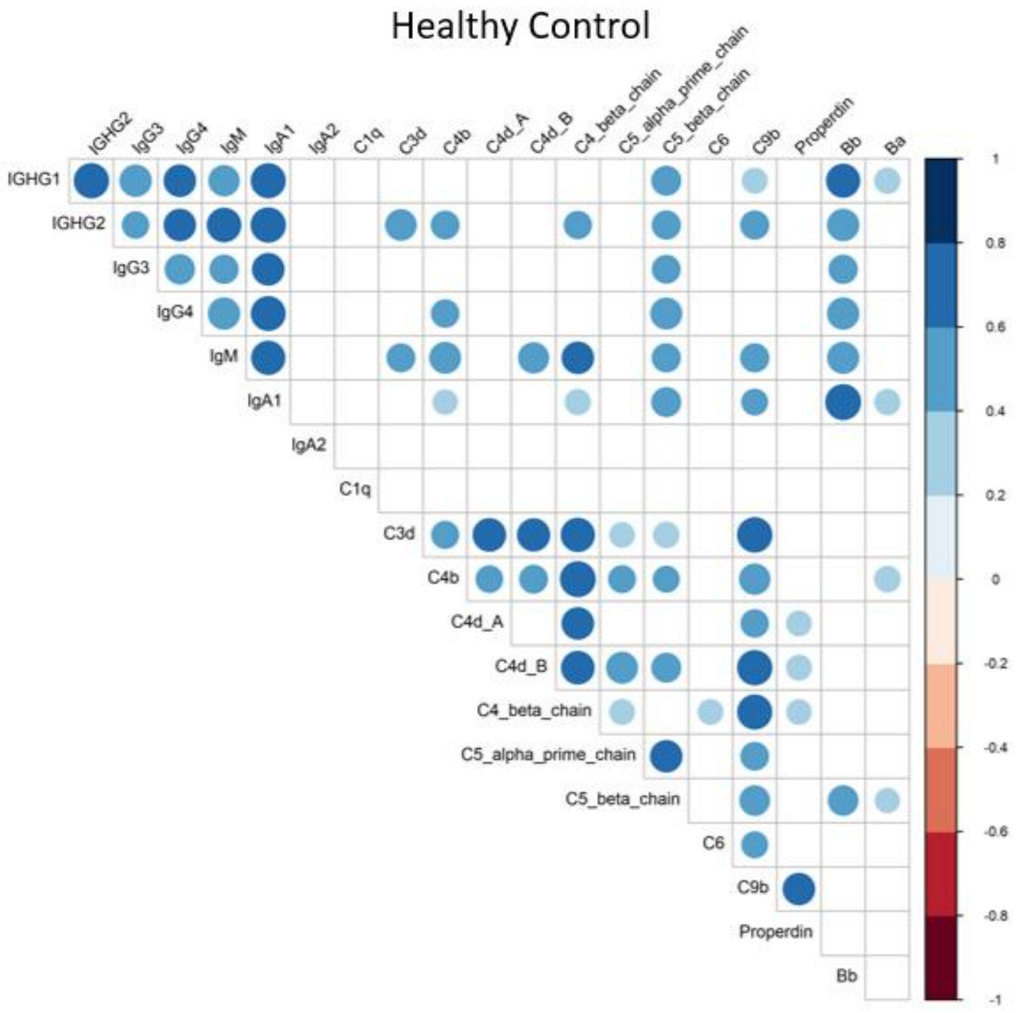

Supplementary Figure 1. Spearman's correlation analyses in healthy control samples revealed no significant relationship between ADA binding and any complement component; however, a strong positive correlation was noted among the complement proteins themselves.

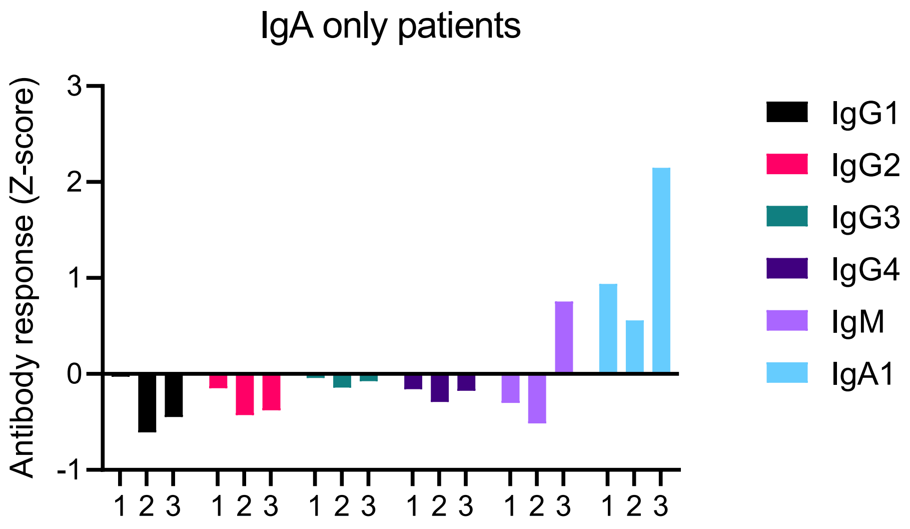

Supplementary Figure 2. Z-Score analysis of three Fabry patients serum demonstrates that some patients present with an IgA1-only phenotype, suggesting a role for serum IgA1 in the developed immune response to ERT.

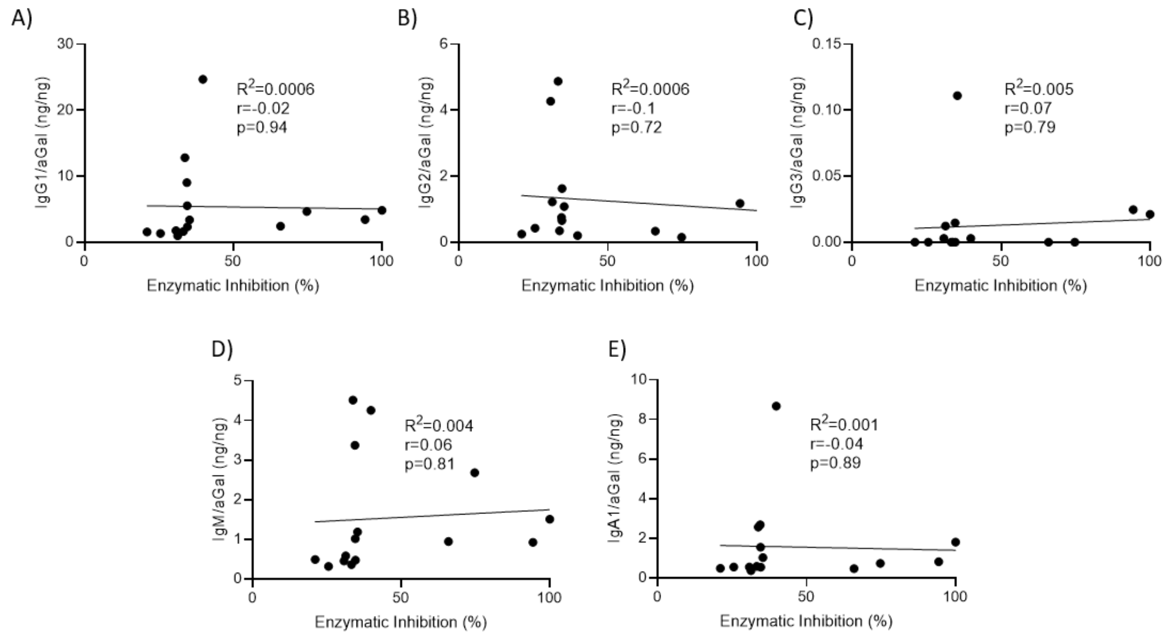

Supplementary Figure 3. Correlation analysis of individual antibody isotype and subclass and direct enzymatic inhibition in Fabry males. No significant correlation was observed against A) IgG1 (p=0.94), B) IgG2 (p=0.72), C) IgG3 (p=0.79), D) IgM (p=0.81) or E) IgA1 (p=0.89). Statistical significance determined by Pearson's correlation.

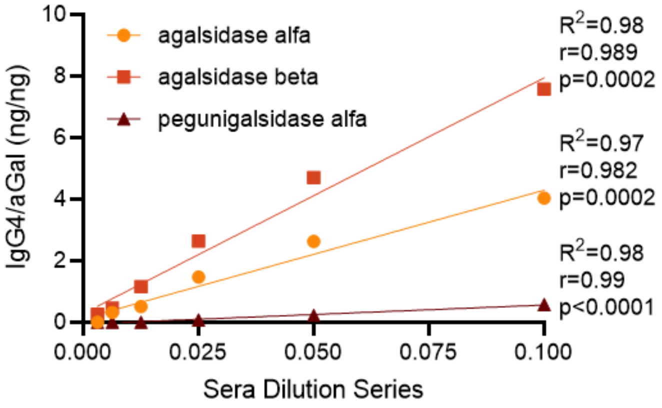

Dilution Series	x=
1 in 10	0.1
1 in 20	0.05
1 in 40	0.025
1 in 80	0.0125
1 in 160	0.006
1 in 320	0.003

Supplementary Figure 4. Correlation analysis of anti-ERT IgG4 in serially diluted sera against each commercially available ERT in a Fabry male positive for ADAs. Significant relationships were observed against agalsidase alfa ($p=0.0002$), agalsidase beta ($p=0.0002$) and pegunigalsidase alfa ($p<0.0001$), demonstrating the specificity of the signal. Statistical significance determined by Pearson's correlation.

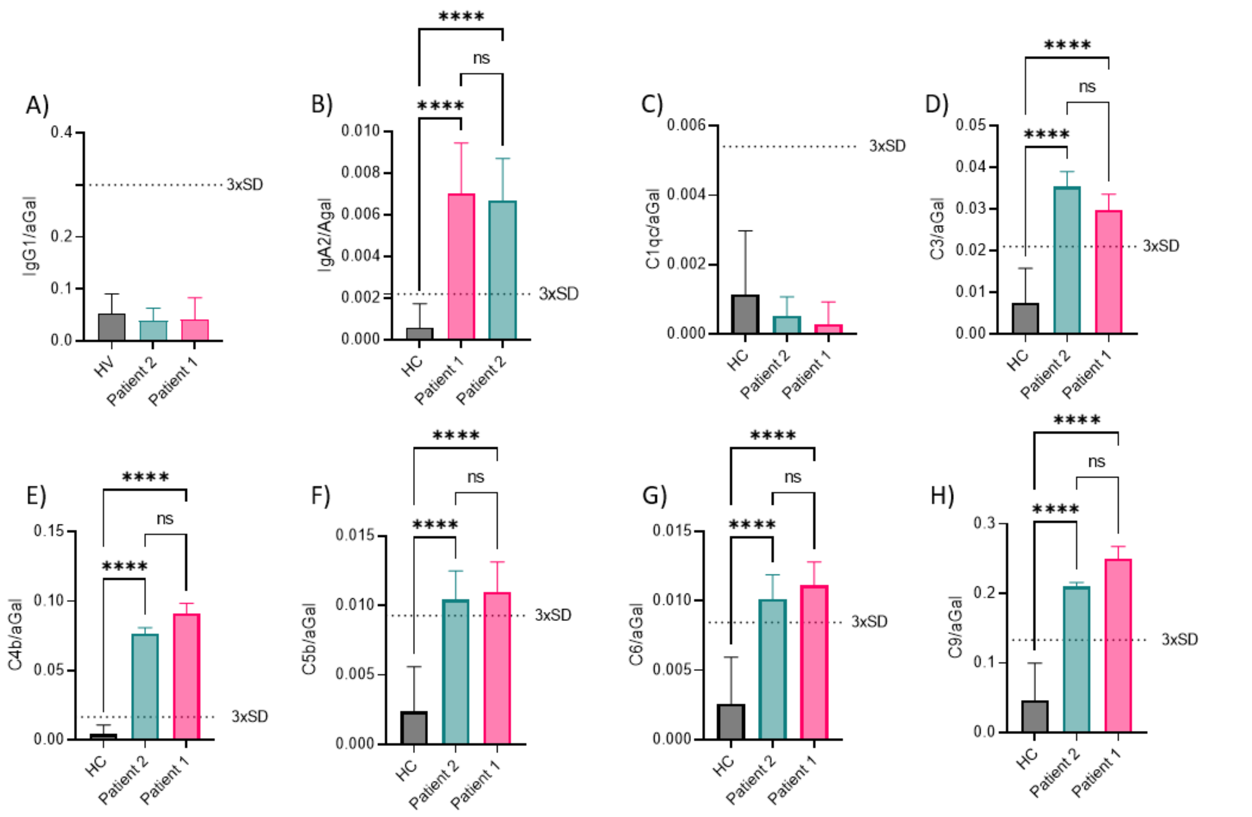

Supplementary Figure 5. Antibody isotype and subclass analysis of two female Fabry patients with intestinal complications post ERT infusion. No significant differences were observed in the levels of A) anti-ERT IgG1, IgG2-4, IgM or IgA1 (data not shown). B) Anti-ERT IgA2 levels were increased in both patients ($p<0.0001$). Whilst there was no observed difference in the levels of C) C1Qc binding, we detected significantly increased levels of later complement components, including D) C3 ($p<0.0001$), E) C4b ($p<0.0001$), F) C5b ($p<0.0001$), G) C6 ($p<0.0001$) and H) C9 ($p<0.0001$). n=8 technical replicates for patient 1 and patient 2, compared to healthy control cohort (n=87 biological replicates). Statistical significance determined by non-parametric ANOVA (Kruskal-Wallis), results considered significant if $p<0.05$.
